# A REAL-WORLD PHARMACOVIGILANCE STUDY OF SACUBITRIL\VALSARTAN IN OLDER PEOPLE

**DOI:** 10.1093/geroni/igae098.0425

**Published:** 2024-12-31

**Authors:** Zheng Kuai, Yu Hu

**Affiliations:** Zhongshan Hospital, Fudan University, Shanghai, Shanghai, China (People’s Republic); Zhongshan Hospital, Fudan University, Shanghai, Shanghai, China (People’s Republic)

## Abstract

**Background:**

Sacubitril\valsartan is the first-in-class angiotensin receptor neprilysin inhibitor which is utilized clinically in the treatment of heart failure with reduced ejection fraction and hypertension patients. However, Systematic studies of sacubitril\valsartan-related adverse events (AEs) signals in older people based on real-world and international databases are still lacking.

**Methods:**

Disproportionality analyses, including the reporting odds ratio (ROR), the proportional reporting ratio (PRR), the Bayesian confidence propagation neural network (BCPNN), and the multi- item gamma Poisson shrinker (MGPS) algorithms, were employed to quantify the signals of Sacubitril\valsartan-associated AEs in older people.

**Results:**

The search retrieved 94,210 sacubitril\valsartan-associated cases within the reporting period; 86 PTs with significant disproportionality were retained in 65+. Angioedema, hypotension, renal impairment, hyperkalemia, cough, dizziness, and seasonal allergies were found at a similar rate to those reported in trials. Reports emerged for several cognitive-related AEs and several fragility acceleration AEs, which were rare. Unexpected safety signals such as ‘Injury, poisoning, and procedural complications’ were only detected in 65+. Most of the cases occurred within the first month after sacubitril\valsartan initiation, and this was consistent across age groups.

**Conclusions:**

Our study found potential new AEs signals and might provide important support for clinical monitoring and risk identification of Sacubitril\valsartan in older people.

